# Supplementary figures and images for: Identification of Novel Metabolic Subtypes Using Multi-Trait Limited Mixed Regression in the Chinese Population
Source: Biomedicines. 2022 Dec 1;10(12):3093. doi: 10.3390/biomedicines10123093 (PMC9775185; doi:10.3390/biomedicines10123093)

**Supplementary figure S1. Cross-validated likelihood for different number of subtypes (K)**

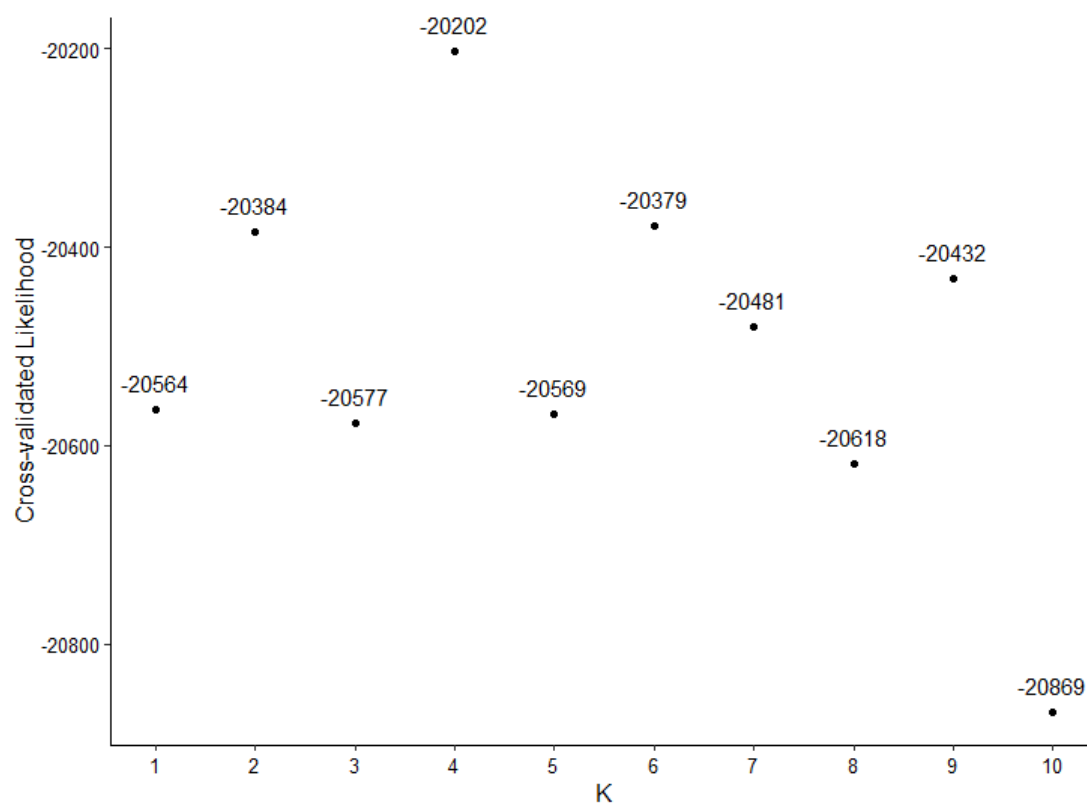

Supplement: Supplementary file 1 [file biomedicines-10-03093-s001.zip › Supplementary figure S1.pdf]
